# Laparoscopic Resection for Ileocecal Intussusception Caused by an Inflammatory Fibroid Polyp: A Case Report

**DOI:** 10.7759/cureus.95799

**Published:** 2025-10-31

**Authors:** Hiroyuki Hazama, Kazumasa Nakamura, Kohei Koido, Takeshi Oshima, Kou Ohata

**Affiliations:** 1 Gastrointestinal Surgery, Shizuoka General Hospital, Shizuoka, JPN

**Keywords:** adult intussusception, gastrointestinal benign tumor, ileocolic resection, inflammatory fibroid polyp, laparoscopic surgery, small bowel obstruction, terminal ileum

## Abstract

Inflammatory fibroid polyp (IFP) is a rare benign mesenchymal tumor of the gastrointestinal tract, typically found in the stomach, while occurrence in the small intestine is uncommon. Adult ileocecal intussusception caused by an IFP is extremely rare. We report the case of a 56-year-old woman who presented with abdominal pain. Abdominal ultrasonography demonstrated a target sign, and contrast-enhanced computed tomography (CT) revealed ileocecal intussusception with a mesenteric vessel converging sign. Colonoscopy identified a protruding lesion with shallow ulceration in the terminal ileum, and biopsy suggested IFP with marked eosinophilic infiltration, although malignancy could not be excluded. The patient underwent laparoscopic ileocecal resection with D3 lymphadenectomy. Histopathological examination confirmed IFP, showing spindle cell proliferation with abundant eosinophilic infiltration and rich vascularization. Immunohistochemically, the lesion was positive for CD34 and negative for c-kit and DOG1. The postoperative course was uneventful, and the patient was discharged on postoperative day 6. IFP should be considered in the differential diagnosis of adult intussusception in the ileocecal region, and laparoscopic oncologic resection represents an appropriate treatment option when malignancy cannot be ruled out.

## Introduction

Inflammatory fibroid polyp (IFP) is a rare benign mesenchymal tumor of the gastrointestinal tract, most frequently occurring in the stomach, while approximately 20% of cases arise in the small intestine [1]. Histologically, IFP is characterized by spindle cell proliferation with prominent eosinophilic infiltration and the concentric arrangement of fibroblasts around blood vessels [2]. The etiology remains uncertain, although somatic mutations in the platelet-derived growth factor receptor alpha (PDGFRA) gene have been identified in many cases, supporting a neoplastic origin rather than a reactive process [3]. The clinical manifestations of IFP vary according to its location within the gastrointestinal tract. Gastric lesions are often asymptomatic or incidentally discovered, whereas small intestinal IFPs can cause abdominal pain, bleeding, or bowel obstruction secondary to intussusception. Because IFPs are benign with no reported malignant potential, complete surgical or endoscopic resection is generally curative. Among small intestinal IFPs, the ileum is the most common site; however, an IFP in the terminal ileum prolapsing into the cecum and causing intussusception is exceedingly rare [4]. Herein, we report an adult case of ileocecal intussusception caused by an IFP that was successfully treated with laparoscopic resection, together with a brief review of the literature.

## Case presentation

The patient was a 56-year-old woman, 150.7 cm in height and weighing 43.3 kg (BMI 19.07 kg/m²). Her Eastern Cooperative Oncology Group performance status was 0, and her American Society of Anesthesiologists physical status was 1. She had a history of dyslipidemia and constipation since the age of 51. Medications included rosuvastatin calcium, ethyl icosapentate, magnesium oxide, mosapride citrate, rebamipide, and rabeprazole sodium. She had no history of smoking, alcohol consumption, transfusion, or allergy.

**Figure 1:**
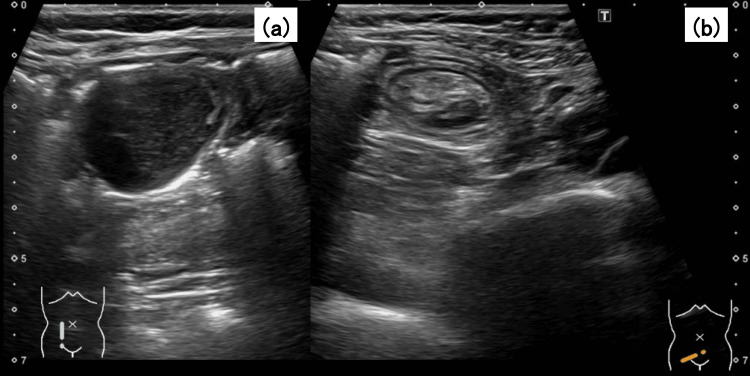
Abdominal ultrasonography: (a) A well-circumscribed, hypoechoic mass measuring 28×23×22 mm was identified in the right lower quadrant. (b) A "target sign" characteristic of ileocecal intussusception was observed

She presented with intermittent, dull upper abdominal pain that had persisted for approximately one week. The pain was mild to moderate in intensity, was not related to meals, and was unaccompanied by nausea, vomiting, or changes in bowel habits. Abdominal ultrasonography revealed a mass and target sign in the right lower abdomen, suggesting intussusception, and she was referred to our department (Figure 1).

**Figure 2:**
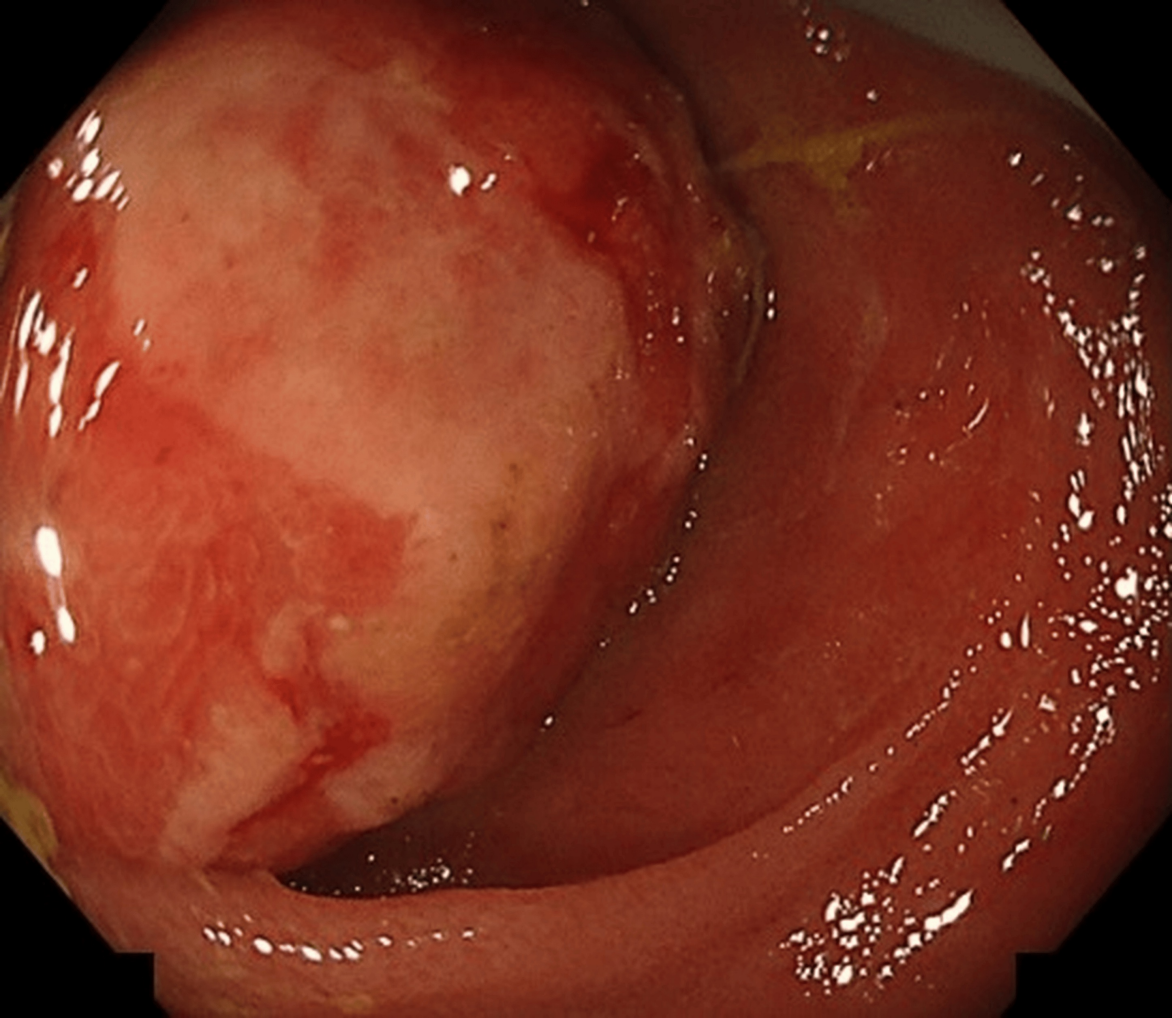
Colonoscopy: A reddish protruding lesion with shallow ulceration located 5 cm proximal to the ileocecal valve, occupying the lumen and preventing passage of the scope

On admission, her abdomen was flat and soft with no tenderness. Laboratory tests, including tumor markers (carcinoembryonic antigen (CEA) 2.3 ng/mL and CA19-9 7 U/mL), were within normal limits (Table 1). Colonoscopy demonstrated a reddish protruding lesion with shallow ulceration located 5 cm proximal to the ileocecal valve. The lesion occupied the lumen, preventing endoscope passage, was firm on probing, and was negative for the cushion sign (Figure 2). Eight biopsy samples were obtained from the ulcerated area.

**Table 1:** Laboratory findings on admission: Mild elevations of AST and ALT were observed; other parameters were within normal ranges AST: aspartate aminotransferase; GOT: glutamic oxaloacetic transaminase; ALT: alanine aminotransferase; GPT: glutamate-pyruvate transaminase; ALP: alkaline phosphatase; γ-GTP: gamma-glutamyl transpeptidase; CEA: carcinoembryonic antigen

Parameter	Result	Reference range	Interpretation
White blood cell count	5,400/μL	3,500-9,000 /μL	Normal
Hemoglobin	13.0 g/dL	11.5-15.0 g/dL	Normal
Platelet count	29.0×10⁴/μL	15.0-35.0×10⁴/μL	Normal
Total protein	7.7 g/dL	6.6-8.1 g/dL	Normal
Albumin	4.3 g/dL	3.8-5.2 g/dL	Normal
AST (GOT)	41 U/L	13-33 U/L	High
ALT (GPT)	35 U/L	6-30 U/L	High
ALP	68 U/L	115-359 U/L	Normal
γ-GTP	17 U/L	10-47 U/L	Normal
Total bilirubin	0.6 mg/dL	0.3-1.2 mg/dL	Normal
Blood urea nitrogen	12 mg/dL	8-20 mg/dL	Normal
Creatinine	0.66 mg/dL	0.46-0.79 mg/dL	Normal
Sodium	145 mEq/L	138-145 mEq/L	Normal
Potassium	4.6 mEq/L	3.6-4.8 mEq/L	Normal
Chloride	108 mEq/L	101-108 mEq/L	Normal
C-reactive protein	0.06 mg/dL	<0.3 mg/dL	Normal
CEA	2.3 ng/mL	<5.0 ng/mL	Normal
CA19-9	7 U/mL	<37 U/mL	Normal

**Figure 3:**
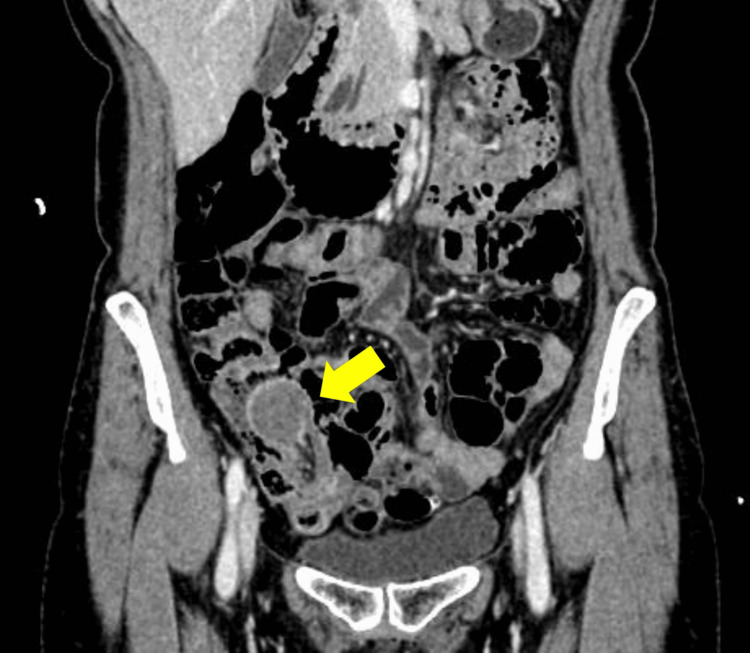
Contrast-enhanced CT (lead mass): A spherical mass (arrow) in the terminal ileum prolapsing into the ascending colon CT: computed tomography

**Figure 4:**
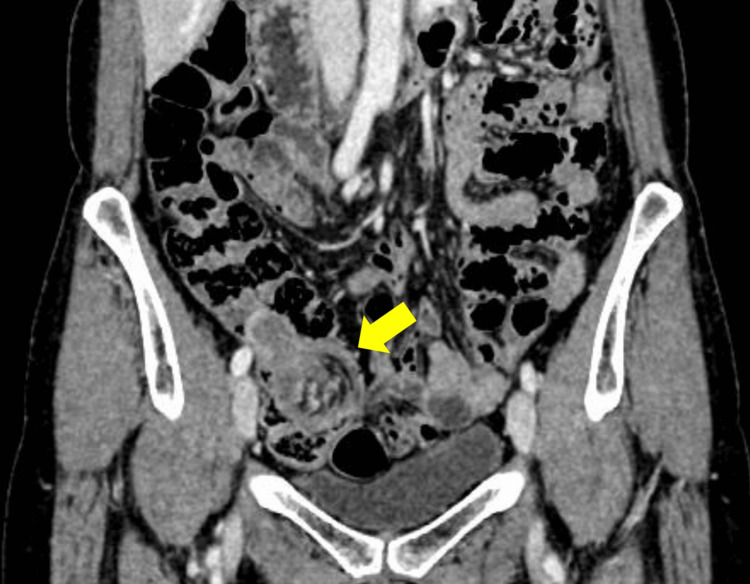
Contrast-enhanced CT (mesenteric vessel converging sign): Mesenteric vessels converging toward the lead mass (arrow) CT: computed tomography

Contrast-enhanced abdominal computed tomography (CT) showed a well-circumscribed, homogeneously enhancing spherical mass approximately 30 mm in diameter in the right lower abdomen, with the ileum prolapsing into the ascending colon. A mesenteric vessel converging sign was also observed (Figures 3-4).

**Figure 5:**
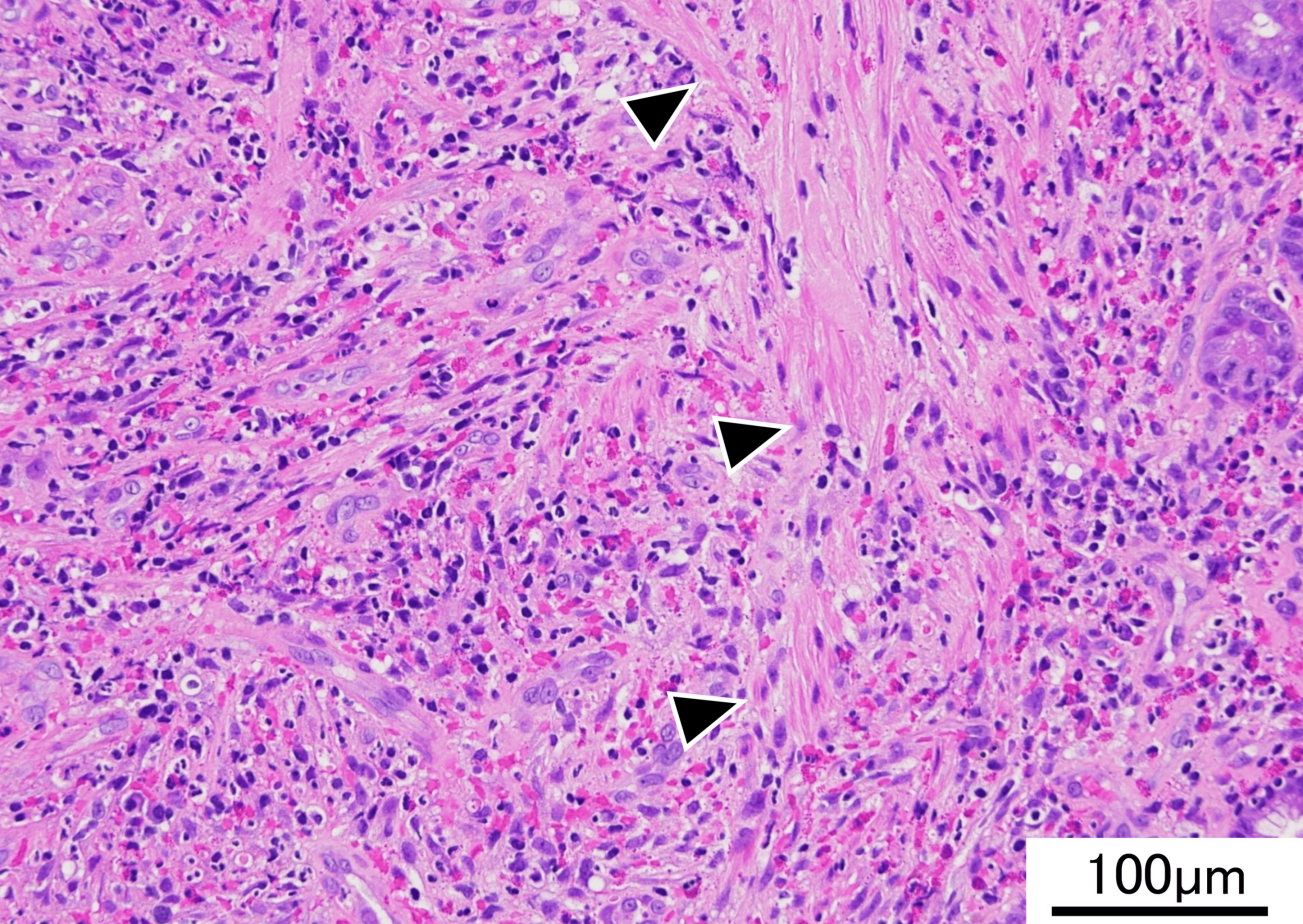
Histopathological findings of the biopsy specimen: Hematoxylin and eosin staining of the biopsy specimen from the terminal ileum shows the granulation tissue with spindle cell proliferation and marked eosinophilic infiltration. The right half of the figure shows the lamina propria and the muscularis mucosae (arrowheads)

Histological examination of the biopsies revealed ulcer formation with fibrous granulation tissue, regenerative mucosa, and marked eosinophilic infiltration. Based on these findings, IFP was considered as one of the possible differential diagnoses, although the lesion could not be definitively diagnosed on biopsy (Figure 5). However, small bowel carcinoma (cT2N0M0, stage I, Union for International Cancer Control (UICC) Eighth Edition) could not be excluded because the endoscope could not pass the lesion, making adequate biopsy sampling difficult. Moreover, given that most small bowel tumors are malignant, carcinoma could not be completely ruled out. Therefore, laparoscopic ileocecal resection with regional lymphadenectomy was planned as a diagnostic and therapeutic procedure.

**Figure 6:**
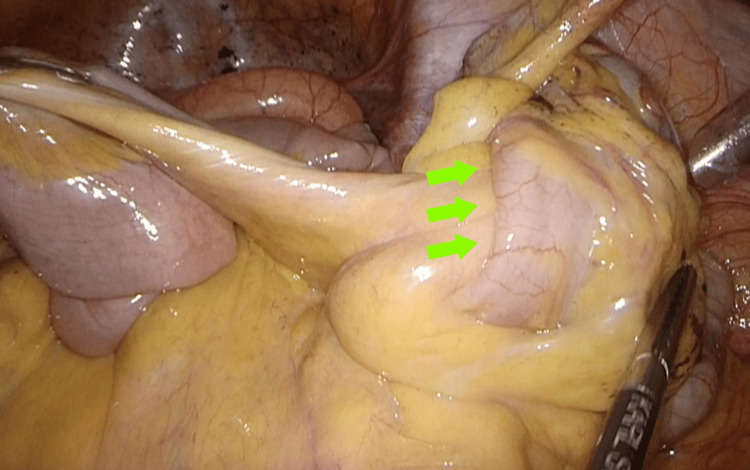
Intraoperative findings: The ileum invaginated into the ascending colon across the ileocecal valve (arrow), with the tumor as the lead point of intussusception

Laparoscopic ileocecal resection was performed with en bloc ligation of the ileocolic vessels at their root, followed by standard regional lymphadenectomy. Perfusion at the anastomotic site was not specifically evaluated. A functional end-to-end anastomosis was created using linear staplers. The operative time was two hours and 30 minutes, and blood loss was 7 mL. Intraoperatively, the ileum was invaginated into the ascending colon across the ileocecal valve, with the tumor serving as the lead point of intussusception (Figure 6). Reduction was not attempted, and resection was completed.

**Figure 7:**
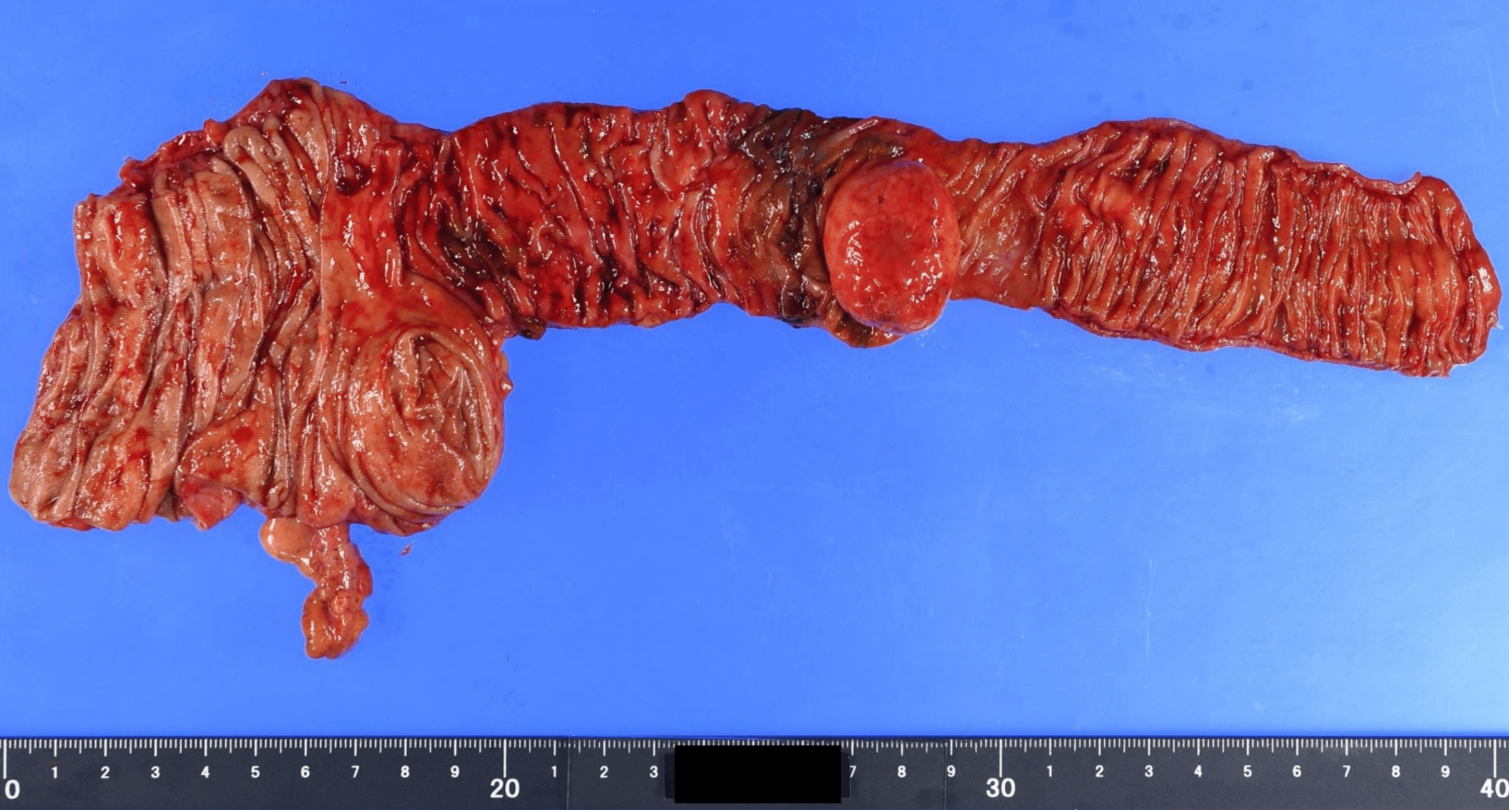
Resected specimen: A spherical tumor, 3 cm in diameter, located at the terminal ileum, filling the lumen and serving as the lead point of intussusception

**Figure 8:**
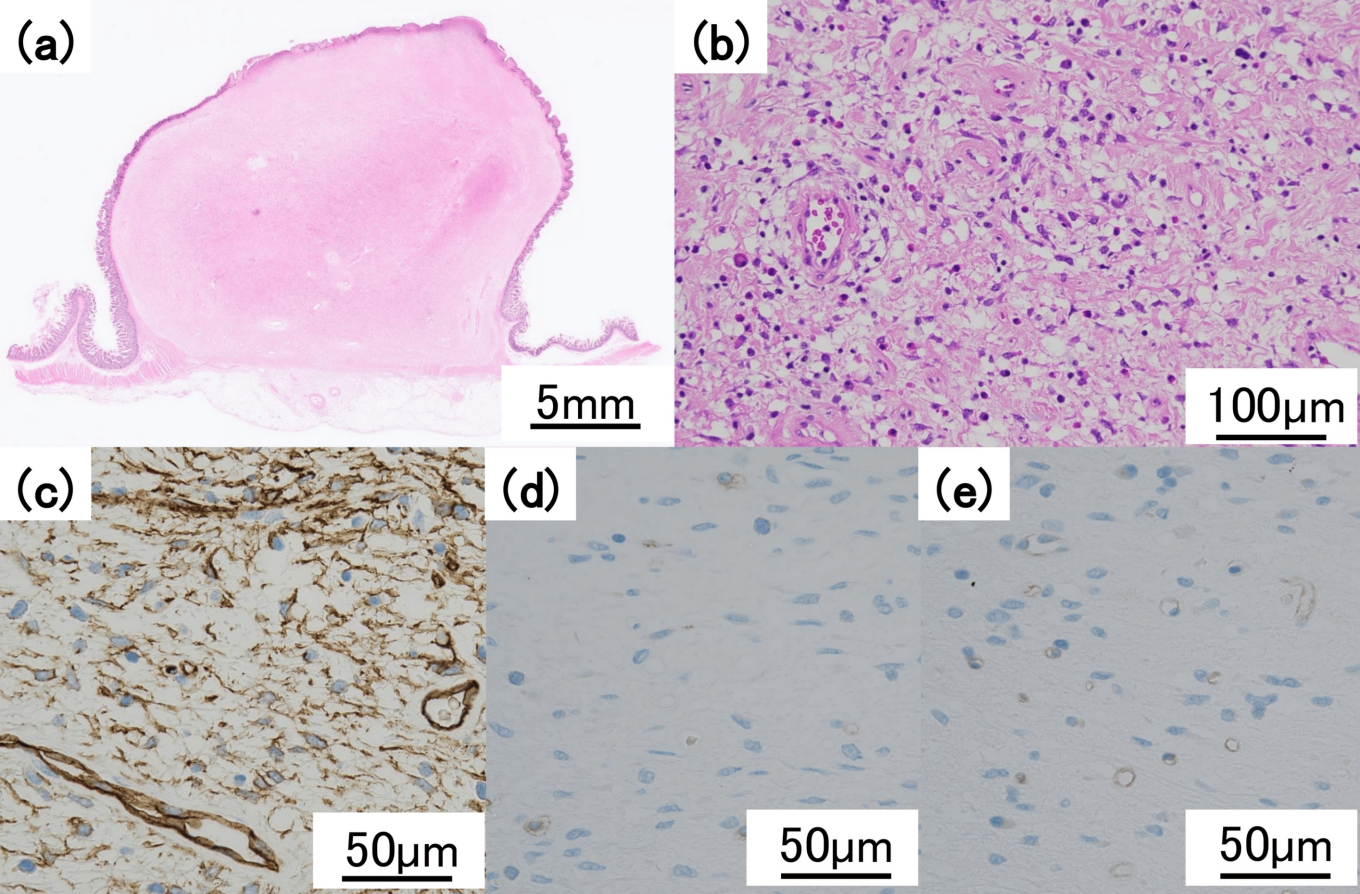
(a) Hematoxylin and eosin stain, low magnification: A well-circumscribed submucosal tumor. (b) Hematoxylin and eosin stain, high magnification: The tumor showed spindle cell proliferation, eosinophilic infiltration, and abundant angiogenesis. (c) Immunohistochemistry: CD34 positivity. (d) Immunohistochemistry: c-kit negativity. (e) Immunohistochemistry: DOG1 negativity

The postoperative course was uneventful, and the patient was discharged on postoperative day 6. The resected specimen revealed a spherical mass measuring 3 cm at the terminal ileum (Figure 7). Histopathological examination revealed mild spindle cell proliferation with prominent eosinophilic infiltration and rich vascularization. Immunohistochemically, the lesion was positive for CD34 and negative for c-kit and DOG1. Additional stains for desmin, αSMA, S100, and SOX10 were all negative, excluding other differential diagnoses such as leiomyoma, schwannoma, granular cell tumor, and inflammatory myofibroblastic tumor (IMT) (Figure 8). A definitive diagnosis of IFP was established. The postoperative course was uneventful. The patient resumed oral intake on postoperative day 3 and was discharged on postoperative day 6 without complications. Follow-up at three months postoperatively revealed no recurrence or complications, and she remained asymptomatic thereafter.

## Discussion

IFP is a rare benign mesenchymal tumor of the gastrointestinal tract, most commonly affecting the stomach, with the small intestine accounting for only about 20% of cases [1]. Within the small intestine, the ileum is the most frequent site. Intussusception of an IFP into the cecum, as in the present case, is exceedingly rare. In a systematic review of 77 small bowel IFP cases, Ivaniš et al. reported that 78% arose from the ileum, with abdominal pain and intussusception as the main symptoms, and more than 92% of patients underwent surgical resection [2]. Other rare reports include giant IFPs causing intussusception and duodenal lesions, but these remain sporadic [3,4]. This case is notable not only for its rarity but also for illustrating the diagnostic uncertainty and treatment decision-making in adult intussusception caused by a submucosal ileal lesion.

Adult intussusception accounts for only 5-10% of all intussusceptions and 1-5% of bowel obstructions [5,6]. Unlike pediatric cases, more than 90% of adult cases are caused by organic lesions, and in the colon, 30-65% are associated with malignancy [5,7]. Accordingly, when malignancy cannot be confidently excluded, simple reduction is generally insufficient, and oncologic resection is recommended. In our case, preoperative exclusion of malignancy was not feasible; therefore, laparoscopic ileocecal resection with D3 lymphadenectomy was performed in accordance with oncologic principles and prior reports [8].

From a diagnostic standpoint, ultrasonography and contrast-enhanced CT revealed a well-circumscribed, homogeneously enhancing mass with a bowel-within-bowel configuration and a mesenteric vessel converging sign, facilitating the diagnosis of intussusception but not allowing a definitive distinction between benign and malignant lesions. Colonoscopy identified a protruding ulcerated lesion; however, the endoscope could not traverse the narrowed lumen, and only limited biopsies from the ulcerated surface could be obtained. Histology showed ulceration with fibrous granulation tissue, regenerative mucosa, and marked eosinophilic infiltration, findings that suggested IFP but did not conclusively exclude neoplasia due to the limited sampling of the submucosal component. Preoperative definitive diagnosis of IFP is uncommon, as histopathological confirmation after resection is often required [9,10].

Regarding treatment options, endoscopic or limited wedge resection may be feasible when IFP is confidently diagnosed preoperatively and the lesion is accessible. In the present case, these approaches were impractical because the 30-mm submucosal lesion in the terminal ileum caused intussusception and near-complete luminal obstruction, precluding safe endoscopic manipulation and adequate submucosal dissection. Furthermore, limited resection without lymphadenectomy risked undertreating a potential malignancy, given the inability to obtain representative submucosal tissue preoperatively. For these reasons, a diagnostic-therapeutic oncologic resection with central ligation (D3 lymphadenectomy) was selected to ensure complete removal and accurate staging. This strategy aligns with previous recommendations favoring oncologic resection in adult intussusception when malignancy cannot be excluded [8-10].

Pathologically, this case demonstrated spindle cell proliferation with abundant eosinophilic infiltration and rich vascularization. A concentric perivascular arrangement ("onion-skin" pattern), which is occasionally observed in IFPs, was focally present. Immunohistochemically, the lesion showed CD34 positivity and was negative for c-kit and DOG1, distinguishing it from gastrointestinal stromal tumor (GIST). IMT was also considered as a differential diagnosis; however, marked eosinophilic infiltration on hematoxylin-eosin staining and the absence of desmin and αSMA expression in spindle cells were inconsistent with IMT, supporting the diagnosis of IFP. Although once regarded as reactive, many IFPs harbor activating PDGFRA mutations, supporting their clonal benign nature. Compared with GIST, these mutations exhibit weaker downstream signaling, consistent with the indolent behavior of IFP [3,11,12].

In summary, this case represents an exceedingly rare IFP of the terminal ileum prolapsing into the cecum and causing intussusception. When malignancy cannot be excluded due to limitations in preoperative tissue sampling and endoscopic access, oncologic resection remains the most appropriate treatment. IFP should be considered in the differential diagnosis of adult intussusception, with management tailored to diagnostic certainty, lesion accessibility, and oncologic safety.

## Conclusions

We reported a rare case of ileocecal intussusception caused by an IFP of the terminal ileum. Preoperative diagnosis remains challenging, and IFP should be considered as a differential diagnosis of adult intussusception. When malignancy cannot be ruled out, oncologic resection is the treatment of choice.

## References

[1] Garmpis N, Damaskos C, Garmpi A (2021). Inflammatory fibroid polyp of the gastrointestinal tract: a systematic review for a benign tumor. In Vivo.

[2] Ivaniš N, Tomas V, Vranić L (2020). Inflammatory fibroid polyp of the small intestine: a case report and systematic literature review. J Gastrointestin Liver Dis.

[3] Akbulut S, Sevinc MM, Cakabay B, Bakir S, Senol A (2009). Giant inflammatory fibroid polyp of ileum causing intussusception: a case report. Cases J.

[4] Zlatarov A, Stefanova N, Mihaylov S, Malinova D (2021). Rare finding of inflammatory fibroid polyp of the duodenum: a complete diagnostic and pathological workup. Cureus.

[5] T Chand J, R R, Ganesh MS (2024). Adult intussusception: a systematic review of current literature. Langenbecks Arch Surg.

[6] González-Carreró Sixto C, Baleato-González S, García Palacios JD, Sánchez Bernal S, Junquera Olay S, Bravo González M, García Figueiras R (2023). Intestinal intussusception in adults: location, causes, symptoms, and therapeutic management. Radiologia (Engl Ed).

[7] Marinis A, Yiallourou A, Samanides L, Dafnios N, Anastasopoulos G, Vassiliou I, Theodosopoulos T (2009). Intussusception of the bowel in adults: a review. World J Gastroenterol.

[8] McKay R (2006). Ileocecal intussusception in an adult: the laparoscopic approach. JSLS.

[9] Wang J, Tian X, Ning BF (2020). Clinical characteristics and prognosis of inflammatory fibroid polyp in the gastrointestinal tract: a series of nine cases and a literature review. J Dig Dis.

[10] Harned RK, Buck JL, Shekitka KM (1992). Inflammatory fibroid polyps of the gastrointestinal tract: radiologic evaluation. Radiology.

[11] Guérit E, Arts F, Dachy G, Boulouadnine B, Demoulin JB (2021). PDGF receptor mutations in human diseases. Cell Mol Life Sci.

[12] Huss S, Wardelmann E, Goltz D (2012). Activating PDGFRA mutations in inflammatory fibroid polyps occur in exons 12, 14 and 18 and are associated with tumour localization. Histopathology.

